# Analytical and Structural Evaluation of Recombinant Human Serum Albumin and Fragment F8 for Aptamer-Based Urinary Biomarker Detection

**DOI:** 10.1021/acsomega.5c03518

**Published:** 2025-07-14

**Authors:** Chanya Archapraditkul, Jarunee Vanichtanankul, Thanaya Saeyang, Wireeya Chawjiraphan, Poramin Boonbanjong, Prapasiri Pongprayoon, Deanpen Japrung

**Affiliations:** † Faculty of Science, Department of Chemistry, 54775 Kasetsart University, Chatuchak, Bangkok 10900, Thailand; ‡ 61191 National Center for Genetic Engineering and Biotechnology (BIOTEC), National Science and Technology Development Agency (NSTDA), Thailand Science Park, Pathumthani 12120, Thailand; § National Nanotechnology Center (NANOTEC), National Science and Technology Development Agency (NSTDA), Thailand Science Park, Pathumthani 12120, Thailand; ∥ Program in Translational Medicine, Chakri Naruebodindra Medical Institute, Faculty of Medicine Ramathibodi Hospital, 26687 Mahidol University, Samut Prakan 10540, Thailand; ⊥ Center for Advanced Studies in Nanotechnology for Chemical, Food and Agricultural Industries, KU Institute for Advanced Studies, Kasetsart University, Bangkok 10900, Thailand

## Abstract

Human serum albumin (HSA) is a key biomarker for early detection of kidney disorders such as microalbuminuria. However, its structural complexity and susceptibility to degradation in urine challenge current diagnostic assays, particularly immunoturbidimetric methods that depend on epitope stability. In this study, we expressed and characterized full-length recombinant HSA (rHSA) and its proteolytic fragment F8 (residues 496–585) in both *Escherichia coli* and rice-based systems to explore their suitability for biosensor validation and diagnostic use. Comparative analysis via SDS-PAGE, LC–MS/MS, and dynamic light scattering revealed differences in solubility and aggregation profiles between expression systems, with rice-derived rHSA showing greater resistance to proteolytic degradation. The F8 fragment retained structural integrity and remained detectable in both native and protease-treated samples. Using a previously developed Cy5-labeled ssDNA aptamer-based fluorescence aptasensor, we evaluated binding to rHSA and F8 across a clinically relevant concentration range (0.37–1500 μg/mL). Sigmoidal fluorescence recovery curves with *R* ^2^ > 0.99 were observed for all recombinant proteins. Notably, the aptasensor successfully detected both targets even under conditions mimicking nonfresh urine, highlighting its diagnostic robustness. These findings establish recombinant F8 as a stable, diagnostically relevant biomarker candidate and confirm the practical utility of aptamer-based platforms for albumin detection in diverse testing scenarios.

## Graphical abstract

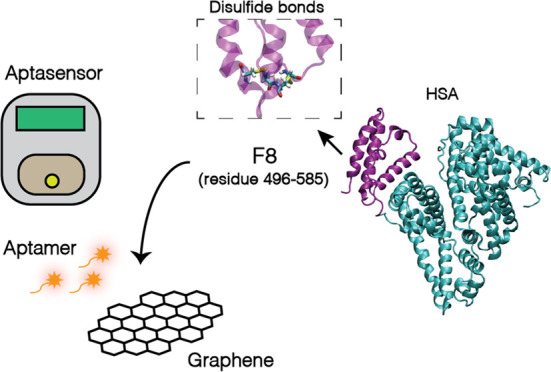

## Introduction

Human serum albumin (HSA) is the most abundant plasma protein and plays a critical role in maintaining oncotic pressure and transporting a variety of endogenous and exogenous molecules.^1^ In clinical diagnostics, HSA is routinely used as a biomarker for kidney function assessment, particularly in detecting microalbuminuria, an early indicator of chronic kidney disease (CKD).^2^ While immunoturbidimetric assays are commonly employed for urinary HSA quantification in hospital laboratories, these antibody-based methods suffer from limitations such as epitope masking in nonfresh samples and variability in antibody binding sites.^3^ Moreover, HSA undergoes proteolytic fragmentation in urine, which may further compromise antibody recognition and reduce diagnostic sensitivity.^4^

Aptamer-based biosensors have emerged as promising alternatives to traditional immunoassays, offering high specificity, stability, and adaptability to complex sample matrices.^5^ Our group previously developed a Cy5-labeled ssDNA aptamer (H8) and graphene oxide (GO)-based fluorescent detection platform capable of sensitively detecting HSA, even in boric acid—preserved or stored urine.^6,7^ However, the use of recombinant albumin and its fragments as analytical targets remains underexplored, particularly regarding their structural characteristics, solubility, and compatibility with aptamer-based sensing.

In this study, we expressed and purified recombinant HSA and its truncated F8 fragment in *Escherichia coli* and rice expression systems. We compared their aggregation behavior and protease susceptibility and evaluated their binding to the H8 aptamer using our established fluorescence-based aptasensor (Figure 1). These results aim to support the broader application of recombinant proteins in biosensor validation and to identify stable albumin-derived fragments such as F8 as potential biomarkers for urinary diagnostics.

**Figure 1:**
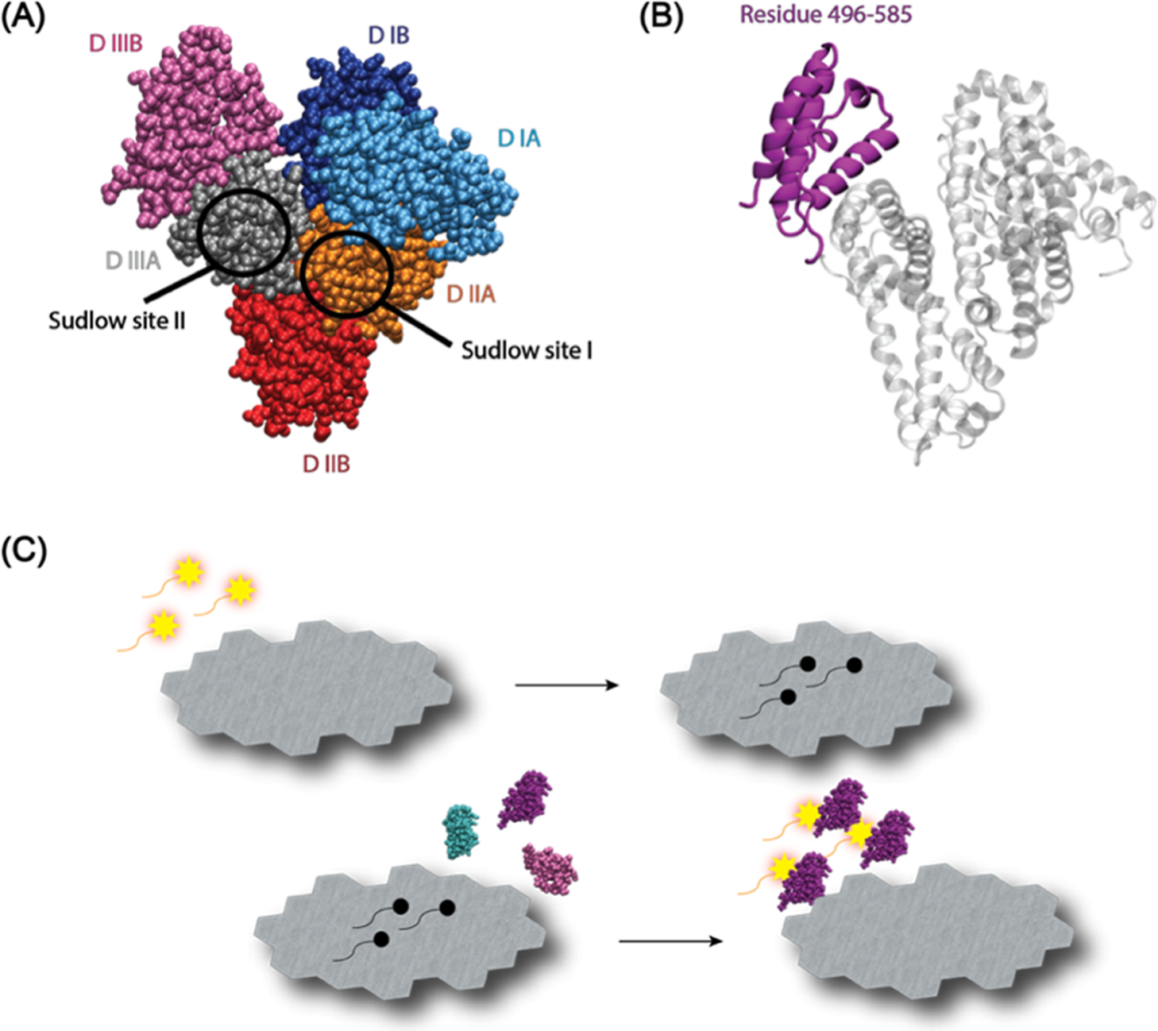
(A) Structural model of human serum albumin (HSA) showing its three-domain architecture (I–III), each subdivided into (A,B) subdomains. The locations of major drug-binding sites are highlighted. (B) Structural representation of fragment 8 (F8), corresponding to residues 496–585 of HSA, shown in purple on the HSA backbone. (C) Schematic of the graphene oxide (GO)-based fluorescence aptasensor principle: fluorescence from the Cy5-labeled aptamer is quenched upon GO binding and restored upon target protein binding and aptamer release.

## Materials and Method

### Materials and Reagents

The rice-derived recombinant HSA was purchased from Sigma-Aldrich (HSA, catalog no. A9731) and protease enzyme (catalog no. P4630) were obtained from Sigma-Aldrich (St. Louis, MO, USA). A lyophilized plasmid encoding the human serum albumin gene (GenBank accession no. AF542069.1) was synthesized by GenScript (New Jersey, USA). Primers for constructing the pET-17b_F8 vector (forward: 5′-TAA GAA GGA GAT ATA CAT ACC ATA TGA CAT ACG TTC CCA AAG AGT-3′; reverse: 5′-GCC AGT GGA TGG ATA TCT GCA GAA TTC TTA AAT GTG ATG TTA GTG-3′) and the 87-nucleotide single-stranded DNA (ssDNA) aptamer labeled with Cy5 (5′-/Cy5/ATA CCA GCT TAT TCA ATT CCC CCG GCT TTG GTT TAG AGG TAG TTG CTC ATT ACT TGT ACG CTC CGG ATG AGA TAG TAA GTG CAA TCT-3′) were synthesized by Integrated DNA Technologies (Singapore). The aptamer was dissolved in DNase/RNase-free water upon receipt and stored at −20 °C until use. Graphene oxide (GO) was synthesized via the modified Hummers method and dissolved in sterile water to a final concentration of 5 mg/mL.^8^

### Degradation of Human Serum Albumin in Artificial Urine

Artificial urine was prepared according to the formulation described in a previous study.^9^ Briefly, 24.3 mg/mL urea was dissolved in distilled water, followed by the addition of 10 mg/mL sodium chloride, 6 mg/mL potassium chloride, and 6.4 mg/mL sodium phosphate. The pH of the solution was adjusted to 6.0–7.0 using 1 N hydrochloric acid (HCl) and 1 N sodium hydroxide (NaOH). A protease enzyme (0.4 unit, pH 7.4) was then added to a 1 mg/mL HSA solution in artificial urine, and the mixture was incubated at 37 °C for 1 h. Protein degradation was subsequently assessed using 12% sodium dodecyl sulfate-polyacrylamide gel electrophoresis (SDS-PAGE) followed by staining with Coomassie Brilliant Blue R-250.

### Protein Identification by LC–MS/MS

Protein bands of interest were excised from SDS-PAGE gels using a sterile surgical blade and subjected to in-gel trypsin digestion.^10^ Proteomic analysis was performed by the Proteomics Services, Faculty of Medical Technology, Mahidol University (Salaya Campus, Nakhon Pathom, Thailand). Gel slices were washed with 50% acetonitrile (ACN) in 25 mM ammonium bicarbonate (NH_4_HCO_3_). Acetonitrile was added to fully reswell the gel pieces and incubated for 10 min, or until the slices appeared opaque white. The solvent was aspirated, and 10–15 μL of 12.5 ng/μL trypsin solution (Promega, USA) was added, followed by overnight incubation at 37 °C (16 h). Peptides were extracted twice by adding 25 μL of 0.1% formic acid (FA) and incubated for 15 min. The resulting peptide mixtures were dried using a nitrogen evaporator (Organomation, USA) and reconstituted in 15 μL of 0.1% FA prior to LC–MS analysis. The LC–MS/MS system consisted of a Dionex Ultimate 3000 RSLCnano system coupled to a Q-ToF Compact mass spectrometer (Bruker, Germany) via a CaptiveSpray ionization source. Peptide separation was performed on an Acclaim PepMap RSLC C18 column (75 μm × 15 cm, nanoViper), protected by a PepMap100 C18 guard column (300 μm × 5 mm, 5 μm particle size). Chromatographic separation was conducted using a linear gradient of 2–85% solvent B (80% ACN, 0.1% FA) over 50 min at a flow rate of 0.3 μL/min. Solvent A consisted of 0.1% FA in water. MS spectra were acquired in positive ion mode over the *m*/*z* range of 300–1500. The most intense peptide ions were selected for MS/MS fragmentation, and the resulting spectra were searched for sequence identification using Mascot Daemon version 2.6.0 (Matrix Science, London, UK).

### Construction of Expression Vectors

The recombinant plasmid pET17b-HSA was constructed by GenScript (New Jersey, USA) within NdeI and *Eco*RI sites (Figure 2A). The F8 gene was amplified by polymerase chain reaction (PCR) from the pET17b-HSA vector using primers described previously, and subsequently cloned into the pET17b, replacing the HSA gene under the control of the T7 promoter (Figure 2B). PCR was carried out using Pfu DNA polymerase (Promega, USA) under the following thermal cycling conditions: initial denaturation at 95 °C for 5 min; 30 cycles of denaturation at 95 °C for 30 s, annealing at 65 °C for 2 min, and extension at 72 °C for 2 min; followed by a final elongation step at 72 °C for 10 min. The PCR product was inserted into pET17b at the NdeI and *Eco*RI sites using HiFi DNA Assembly Master Mix (New England Biolabs, Germany). Recombinant clones were propagated in *E. coli* DH5α and verified by Sanger sequencing.

**Figure 2:**
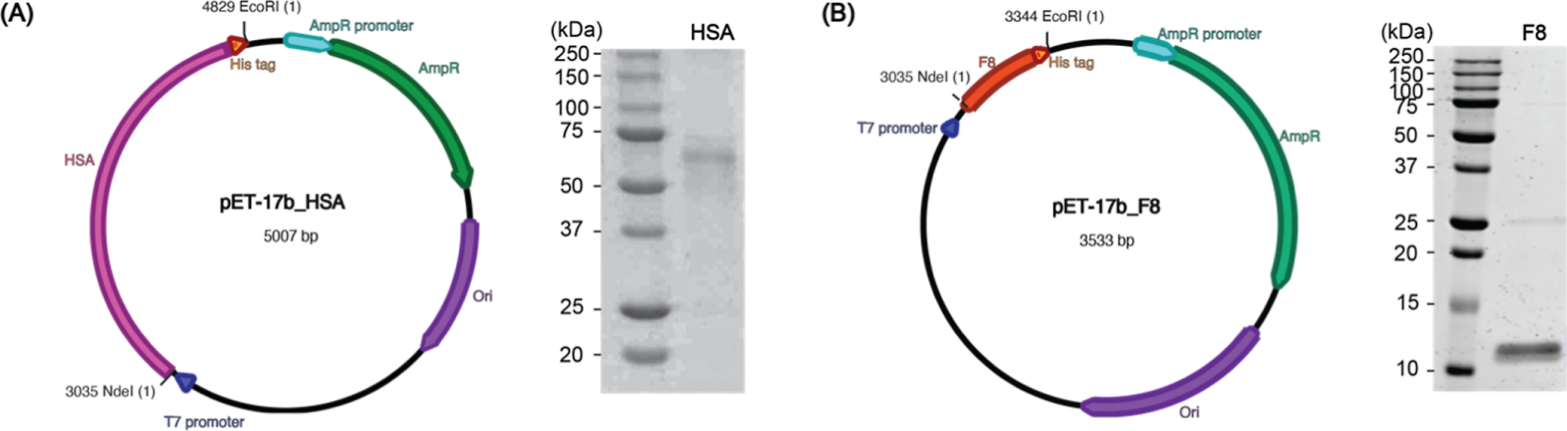
Construction of expression vectors and recombinant protein expression of HSA (A) and F8 (B). The pET17b-HSA and pET17b-F8 plasmids were designed to express His-tagged HSA (67.5 kDa) and F8 (11.0 kDa), respectively, under the control of the T7 promoter. SDS-PAGE analysis confirmed expression of the target proteins in *E. coli*, with HSA and F8 observed in 12.5% and 15% acrylamide gels, respectively.

### Expression of rHSA and rF8

*E. coli* SHuffle T7 Express lysY (NEB) were individually transformed with the recombinant plasmids pET17b-HSA and pET17b-F8. Transformed cells were cultured in Luria–Bertani (LB) medium supplemented with 100 μg/mL ampicillin and 33 μg/mL chloramphenicol at 37 °C until the optical density at 600 nm (OD_600_) reached 0.6. Protein expression was induced with 0.4 mM isopropyl β-D-1-thiogalactopyranoside (IPTG), and cultures were incubated for 16 h at 18 °C with shaking at 200 rpm. Cells were harvested by centrifugation at 4500*g* for 10 min at 4 °C. The resulting cell pellet was resuspended in 50 mL of ice-cold lysis buffer (50 mM Tris–HCl, pH 8.0, containing 100 mM NaCl and 20 mM imidazole). Cell disruption was performed using a French Press (Glen Mills, USA) to extract soluble proteins. The lysate was centrifuged at 15,000*g* for 1 h at 4 °C, and the supernatant was collected for purification. His-tagged proteins were purified using a nickel–nitrilotriacetic acid (Ni–NTA) affinity column (QIAGEN, Germany). Proteins were eluted using a gradient of imidazole (20–500 mM) in elution buffer (50 mM Tris–HCl, pH 8.0, 100 mM NaCl). Eluted fractions were collected, aliquoted, and analyzed by SDS-PAGE. Protein concentrations were determined using the Bradford assay,^11^ and purity was assessed by densitometric analysis using Image Studio 5.2 software (LI-COR Biosciences, USA).

### Dynamic Light Scattering Analysis

Prior to measurement, protein samples were diluted to a final concentration of 350 μg/mL in phosphate-buffered saline (PBS, pH 7.4; Cytiva, Sweden) and filtered through a 0.22 μm poly­(ether sulfone) (PES) membrane (BIOFIL, China). DLS analysis was performed using a Zetasizer Nano ZS instrument (Malvern Panalytical, UK), which includes a laser light source, sample cell, detector, and correlator. Measurements were conducted in 12 mm outer-diameter disposable square polystyrene cuvettes (Malvern Panalytical, UK) containing 2 mL of filtered protein solution. The system was configured with a 4 mW helium–neon (He–Ne) laser at a wavelength (λ_0_) of 633 nm and a scattering angle (θ) of 173°. The intensity-based particle size distribution was recorded for each sample.

### Fluorescence-Based Aptasensor Assay for HSA and F8 Detection

The binding affinity of the graphene oxide (GO)-based fluorescence aptasensor was evaluated in phosphate-buffered saline (PBS, pH 7.4) following a modified protocol from previous studies.^6^ Briefly, 15 μL of 5 mg/mL GO was incubated with 15 μL of 5 μM Cy5-labeled H8 aptamer at room temperature in the dark for 5 min to allow complex formation. After formation of the GO–aptamer complex, various concentrations of human serum albumin (HSA) and its F8 fragment (0.37–1500 μg/mL) were added. The final volume was adjusted to 200 μL and incubated for an additional 30 min under dark conditions. Fluorescence intensity was measured using a Quantus portable fluorometer (Promega Corp., Madison, WI, USA) with an excitation wavelength of 630 nm and emission at 670 nm. Fluorescence recovery (Δ*F*) was calculated using the equation

$$\Delta F=F_{ob}-F_{\min}$$

*F*_ob_ is the fluorescence intensity in the presence of protein and *F*_min_ is the fluorescence intensity of the GO–aptamer complex in the absence of protein. A nonlinear regression model was applied to generate the calibration curves, and the limit of detection (LOD) was calculated using the formula LOD = 3.3 × SD/slope.

## Results and Discussion

### Expression and Purification of rHSA and rF8 in *E. coli*

To enhance the soluble and functional expression of recombinant human serum albumin (rHSA) and its F8 fragment (rF8), *E. coli* SHuffle T7 Express lysY cells were transformed with the pET17b-HSA and pET17b-F8 plasmids, respectively. SHuffle strains facilitate cytoplasmic disulfide bond formation, enabling efficient expression of disulfide-rich proteins such as albumin.^12^ HSA contains 17 disulfide bonds, which are essential for maintaining its structural integrity and function;^1^ SHuffle strains are particularly advantageous in this context as they promote correct disulfide bond formation in the cytoplasm. The F8 gene was amplified by PCR from the pET17b-HSA vector and cloned into the pET17b backbone under the control of the T7 promoter (Figure S1). Following induction and cell harvest, protein expression was analyzed by SDS-PAGE. Prominent bands were observed at 67.5 kDa for rHSA and 11.0 kDa for rF8 in 12.5% and 15% acrylamide gels, respectively (Figure 2). Densitometric analysis revealed high expression purities of 84% for rHSA and 95% for rF8 (Figure S2, Table S2), confirming correct molecular weights and minimal degradation. These high purities are suitable for downstream applications without requiring further purification. The successful, soluble expression of both full-length and truncated HSA demonstrates the effectiveness of the SHuffle expression system for producing structurally complex proteins. Importantly, although the F8 fragment (residues 496–585) lies entirely within subdomain IIIB and retains two native disulfide bonds (Cys514–Cys559 and Cys558–Cys567), its smaller size and simplified structure likely contribute to its higher solubility and efficient expression,^13^ making it highly suitable for binding and sensor studies. This region includes lysine residues known to be glycation-prone, which can induce structural alterations affecting fragment stability and aptamer interaction, as previously reported in our molecular dynamics study.^13^ While post-translational modifications such as glycation or oxidation may occur in native HSA, including within the F8 region, their specific roles in this fragment remain uncharacterized and were not assessed in this study. Furthermore, *E. coli* provides a cost-effective and scalable platform for large-scale production of recombinant HSA fragments, supporting potential diagnostic and biosensor applications.

### Proteolytic Degradation of rHSA and Identification of F8 Fragment

In both healthy and nephrotic urine, serine proteases represent the predominant enzymatic group responsible for protein degradation. To model this in vitro, protease type I from bovine pancreas, which contains a broad range of serine protease activities, was added to artificial urine supplemented with 1 mg/mL of rHSA. In addition to *E. coli*-expressed rHSA, rice-derived rHSA, known to possess glycosylation profiles similar to native human albumin, was included to evaluate the influence of post-translational modifications on protease susceptibility.^14^

After 1 h of incubation, SDS-PAGE analysis revealed substantial degradation of both rice-derived and *E. coli*-derived rHSA proteins, with the characteristic 66 kDa albumin bands no longer clearly visible (Figure 3). Visual inspection of the 12% SDS–PAGE gel indicated a marked reduction in the intensity of the 66 kDa band after protease treatment, accompanied by the appearance of lower molecular weight fragments. The most prominent of these is a ∼10 kDa band, which was consistently observed in both treated rHSA samples and in untreated *E. coli*-derived rHSA. This band closely corresponds to the expected size of the F8 fragment. LC–MS/MS analysis of the excised gel band confirmed 96% sequence similarity to the recombinant F8 peptide (Table S1), validating the identity of this degradation product.

**Figure 3:**
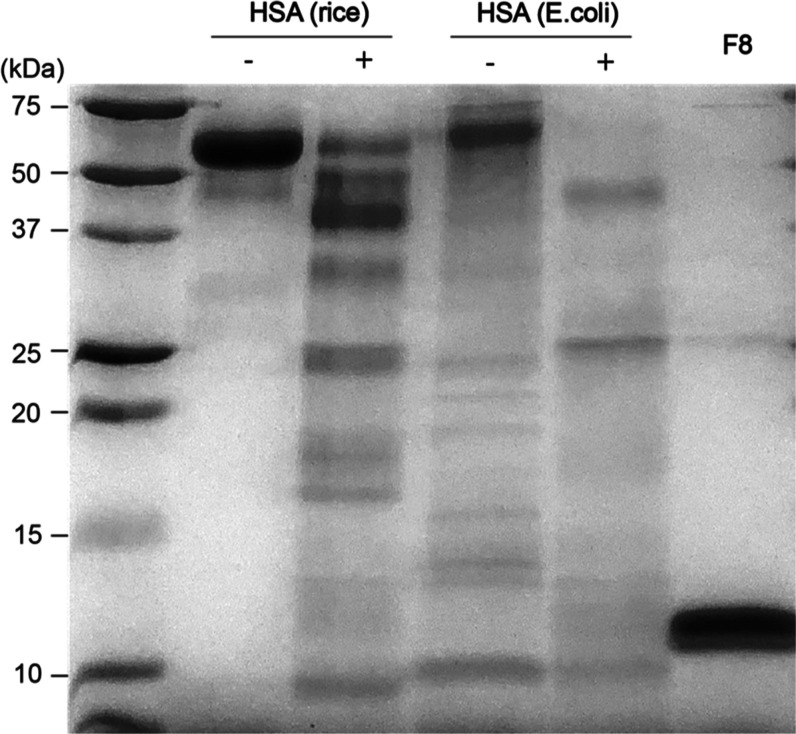
SDS-PAGE analysis of protease-induced degradation of recombinant HSA (rHSA) in artificial urine. Rice- and *E. coli*-derived rHSA samples (1 mg/mL) were incubated with serine protease type I for 1 h at 37 °C. Near-complete degradation of the 66 kDa HSA band was observed in both treated samples. A distinct ∼10 kDa fragment, consistent with the F8 fragment, was detected in both protease-treated samples as well as in untreated *E. coli*-derived rHSA, but was absent in untreated rice-derived rHSA, suggesting glycosylation-dependent resistance to proteolysis.

Interestingly, a similar ∼10 kDa band appeared in the untreated *E. coli*–derived rHSA lane. This band likely reflects a spontaneous degradation product rather than a defined fragment like F8. Due to the absence of post-translational modifications in *E. coli*-derived rHSA, its structural stability is reduced compared to rice-derived rHSA, making it more susceptible to cleavage even in the absence of exogenous proteases.^15^ While the band size is similar to that of F8, it is not necessarily identical, and further sequencing would be required for confirmation, which lies outside the scope of this study.

These findings suggest that glycosylation influences albumin’s susceptibility to degradation, and that F8-like fragments may arise naturally in stored or pathological urine conditions. The presence of the F8 fragment across treatments supports its biological plausibility as a stable urinary biomarker, particularly in chronic kidney disease (CKD), where albumin fragments have been previously identified.^16^

While the rice-derived rHSA exhibited improved resistance to degradation in our artificial urine model, the proteolytic environment in human urine can vary substantially between individuals due to factors such as infection, inflammation, and renal pathology.^4^ Therefore, future studies using patient-derived urine samples are warranted to validate the stability of recombinant albumin variants under diverse clinical conditions.

### Hydrodynamic Size and Aggregation Behavior of rHSA and rF8

Dynamic light scattering (DLS) was used to assess the hydrodynamic diameter and aggregation profile of recombinant HSA and F8 proteins in solution. All measurements were performed in phosphate-buffered saline (PBS, pH 7.4), a physiologically relevant buffer system. While suitable for mimicking in vivo conditions, PBS can also promote protein aggregation in the absence of stabilizing modifications, such as glycosylation. The intensity-weighted size distribution profiles revealed distinct differences between proteins expressed in *E. coli* and those expressed in rice (Figure 4).

**Figure 4:**
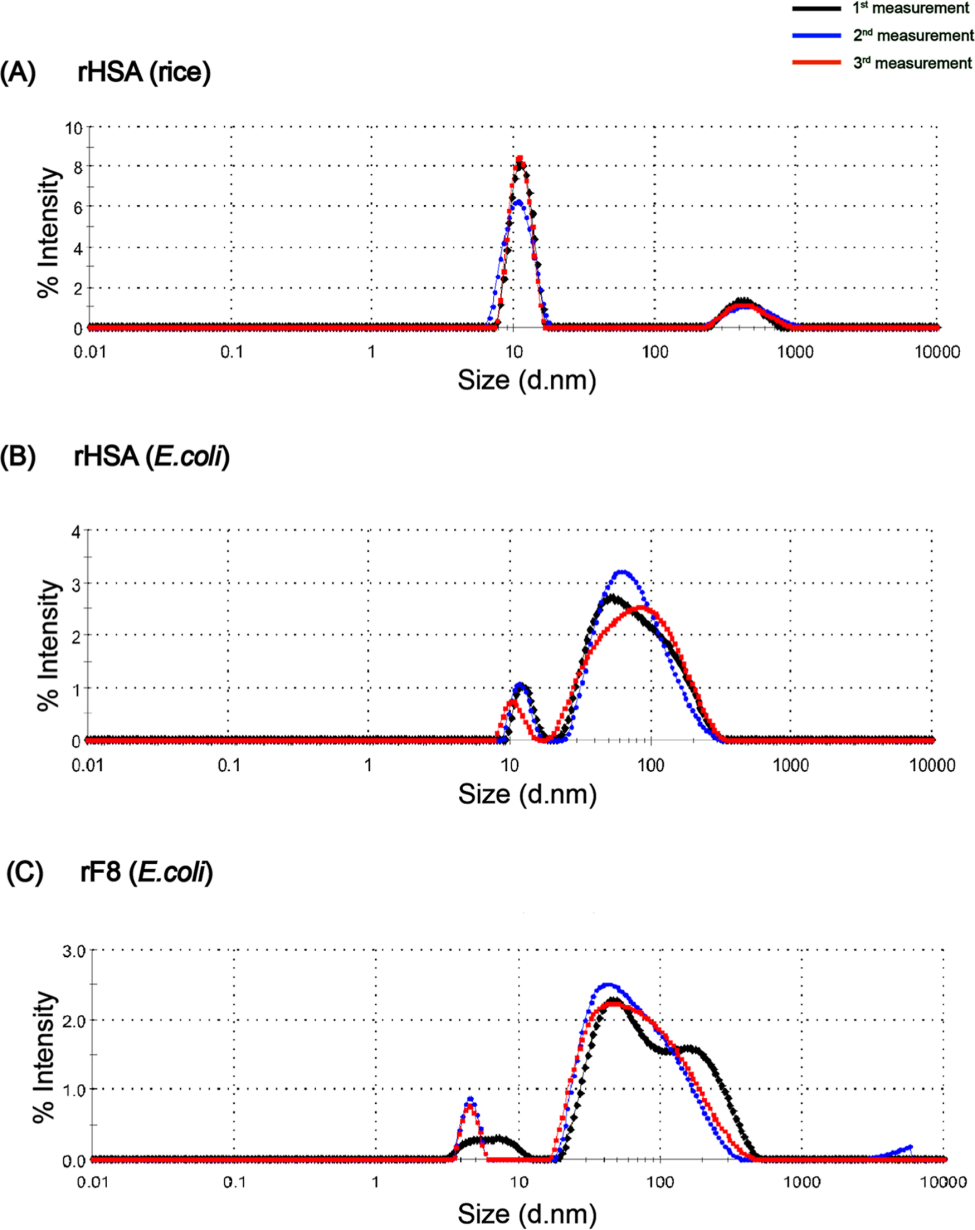
Dynamic light scattering (DLS) analysis of recombinant human serum albumin (rHSA) and F8 fragment (rF8) expressed in different systems. Size distribution by intensity is shown for (A) rHSA expressed in rice, (B) rHSA expressed in *E. coli*, and (C) rF8 expressed in *E. coli*. Rice-derived rHSA exhibited a monodisperse profile centered at ∼11 nm, while *E. coli*-derived rHSA and rF8 displayed broader distributions with dominant peaks above 70 nm, indicating significant protein aggregation.

Rice-derived rHSA exhibited a dominant peak at 11.25 ± 0.26 nm, consistent with previously reported native albumin sizes (Table 1).^17,18^ In contrast, rHSA expressed in *E. coli* showed a higher intensity peak at 86.66 ± 4.64 nm, with a minor peak at 11.85 ± 0.97 nm, indicating a significant presence of aggregates. rF8 expressed in *E. coli* exhibited a similar aggregation trend, with its main peak at 74.19 ± 14.30 nm, a small monomeric peak at 5.24 ± 1.10 nm, and a third peak extending to over 1600 nm, suggesting the formation of large oligomeric species. This was further supported by the polydispersity index (PdI), which exceeded 0.4 for rF8, indicating poor monodispersity and a high degree of aggregation. A PdI below 0.3 is typically indicative of monodisperse, stable protein populations, and the elevated values observed for rF8 confirm its heterogeneous nature.

**Table 1:** Hydrodynamic Size Distribution and Polydispersity Index (PdI) of Recombinant Proteins Measured by DLS^a^

		size (d nm)			area (%)		
sample	PdI	peak 1	peak 2	peak 3	peak 1	peak 2	peak 3
rHSA (rice)	0.33 ± 0.01	11.25 ± 0.26	464.03 ± 23.74		80.20 ± 0.89	19.80 ± 0.89	
rHSA (E. coli)	0.31 ± 0.01	86.66 ± 4.64	11.85 ± 0.97		92.40 ± 1.25	7.60 ± 1.25	
rF8 (E. coli)	0.47 ± 0.02	74.19 ± 14.30	5.24 ± 1.10	1632.73 ± 2653.95	92.83 ± 19.63	5.77 ± 0.49	1.4 ± 0.81

a Values represent mean ± SD for each peak and corresponding intensity-based area percentage.

The observed aggregation in *E. coli*-derived proteins may be attributed to improper folding and reduced stability due to the absence of post-translational modifications. Notably, rice-expressed rHSA exhibited significantly reduced aggregation, likely due to the protective effects of N-glycosylation, which introduces steric hindrance and minimizes intermolecular interactions.^15^ The instability and aggregation of rF8 may also stem from its short sequence length and retention of two disulfide bonds, making it more prone to unfolding in solution. Importantly, the aggregation state of the proteins, particularly in *E. coli*-derived rF8, may influence aptamer binding efficiency, as conformational stability and surface accessibility are critical parameters for aptasensor performance.

While our experimental techniques focused on aggregation and solubility, we acknowledge that detailed conformational and PTM differences are better resolved through structural modeling or biophysical assays. Notably, previous molecular dynamics studies from our group have explored glycation-induced folding changes in HSA and its fragments,^13^ complementing the current experimental findings.^1,2^

Although we did not experimentally assess disulfide bonding or secondary structure by CD spectroscopy, we previously conducted computational modeling that predicted two disulfide bridges within the F8 fragment. A visual representation of this structure is provided as Figure S4 in the Supporting Information. These data support the conformational stability of the fragment and serve as a basis for future experimental validation.

In addition to structural considerations, the choice of expression system also impacts production yield and cost-efficiency. *E. coli* offers rapid growth, low cultivation costs, and high protein yield, particularly for small fragments like F8, making it highly scalable for biosensor applications. In contrast, rice-based systems, while capable of producing glycosylated, native-like albumin, typically involve longer production times and higher costs due to the complexity of plant cultivation and processing.^14,17^ These factors should guide the selection of an expression platform based on application-specific needs.

### Interpretation of Structural Effects for Diagnostic Applications

While our experimental data did not directly assess clinical diagnostic performance, insights from existing literature help contextualize the potential impact of structural differences observed in recombinant HSA from different expression systems. The rice-derived rHSA, which includes post-translational modifications such as glycosylation, showed superior solubility and protease resistance, consistent with previous reports highlighting glycosylation’s stabilizing role in protein folding and resistance to degradation.^15^ These properties suggest that it may be more suitable for diagnostic applications requiring prolonged stability, such as in stored reagent kits or long-term biosensor formats. In contrast, the *E. coli*-derived rHSA, though prone to aggregation and partial fragmentation, demonstrated higher aptamer binding efficiency—likely due to reduced steric hindrance and surface modifications.^14,19^ Therefore, while plant-based systems may better replicate native-like protein structures, bacterial expression systems may still be preferred for biosensor development, particularly when the detection method is robust against conformational variability. The choice of expression host should thus be guided by the intended application, balancing native-like function with manufacturing simplicity and assay sensitivity.^17,18^

### The Performance and Characteristic of rHSA and rF8 Detection by Aptasensor

To evaluate the sensitivity and target binding of the aptasensor system, an 87-nucleotide Cy5-labeled ssDNA aptamer (H8) previously developed for human serum albumin (HSA) recognition was employed.^6,7^ The aptamer was incubated with graphene oxide (GO), which effectively quenched its fluorescence due to π–π stacking interactions.^19^ Upon target addition, fluorescence recovery was observed due to aptamer–protein binding and GO release. The aptasensor was tested across a protein concentration range of 0.37–1500 μg/mL, covering the diagnostic window for microalbuminuria.^2^ The calibration curves generated for each recombinant protein revealed strong sigmoidal correlations between fluorescence intensity and protein concentration, with coefficients of determination (R^2^) exceeding 0.99, indicating excellent curve fitting and high assay precision (Figure 5).

**Figure 5:**
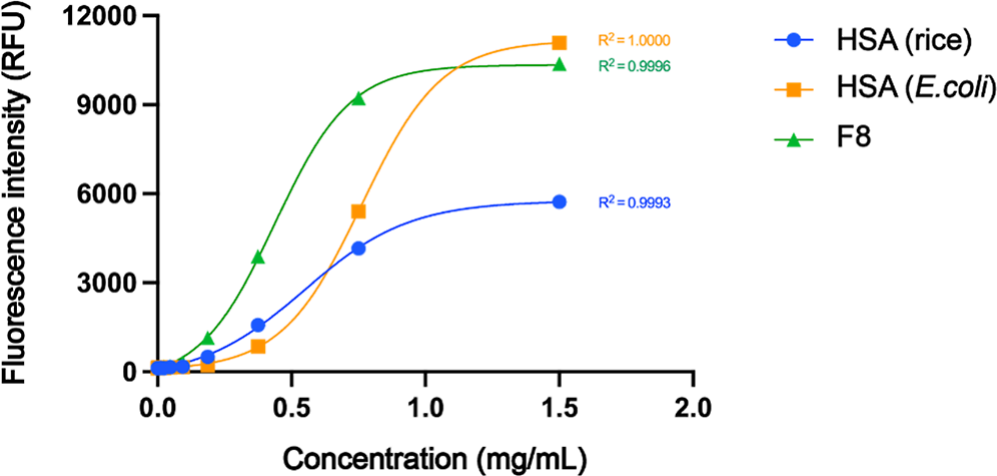
Calibration curves illustrating the fluorescence response of the aptasensor to increasing concentrations of recombinant proteins (0.37–1500 μg/mL). Sigmoidal correlations were observed for rHSA expressed in rice and *E. coli*, as well as for rF8 expressed in *E. coli*, with coefficients of determination (*R* ^2^) exceeding 0.99 in all cases, indicating high sensitivity and curve fitting accuracy.

Our previous aptasensor platform for intact HSA detection utilized graphene oxide–mediated fluorescence quenching under buffer conditions.^6,7^ In comparison, the current fluorescence-labeled aptasensor demonstrates enhanced compatibility with structurally altered or fragmented albumin, such as F8, and maintains performance in protease-spiked artificial urine. This highlights its suitability for real-world diagnostic settings. Other aptasensor platforms have achieved high sensitivity using fluorescence^21,22^ or electrochemical strategies,^23,24^ though often under idealized conditions. Our system offers the advantage of functioning under complex sample conditions without compromising specificity or signal clarity.

Notably, recombinant proteins expressed in *E. coli* (both rHSA and rF8) exhibited higher fluorescence intensities compared to rice-derived rHSA at equivalent concentrations. This discrepancy is likely attributable to the presence of glycosylation in rice-derived HSA, which may introduce steric hindrance and reduce aptamer accessibility to the binding epitope.^15,20^ Interestingly, the F8 fragment generated a comparable fluorescence response to full-length rHSA, suggesting that the aptamer binding site remains accessible and intact within this truncated region.

In contrast to conventional immunoturbidimetric assays, widely used in hospital laboratories, which rely on polyclonal/monoclonal antibodies with poorly defined binding epitopes and are discouraged for use with nonfresh urine due to protein degradation or epitope masking,^3^ the aptasensor developed in this study demonstrated robust detection of recombinant albumin and its fragments under buffered conditions. Moreover, previous work from our group has shown that the same aptasensor retains its performance in boric acid-preserved urine stored under various conditions, including ambient temperature, refrigeration, and multiple freeze–thaw cycles,^7^ highlighting its practical advantage for delayed-sample or field diagnostics.

While this study was conducted in PBS, future validation in clinical urine matrices will be essential to confirm specificity and performance in complex biological environments. Taken together, these findings confirm the compatibility of the F8 fragment and recombinant HSA variants with aptamer-based fluorescence detection. The aptasensor effectively distinguished glycosylated and nonglycosylated forms of albumin, retained performance across a clinically relevant concentration range, and demonstrated robust signal recovery even in conditions simulating degraded samples. These results support the utility of recombinant proteins for aptasensor calibration and highlight F8 as a stable, diagnostically relevant degradation product suitable for future urinary biomarker applications.

In complex biological matrices such as urine, peptide–DNA (aptamer) interactions are influenced by multiple factors including ionic strength, presence of metabolites, competing proteins, and pH. These factors can affect aptamer folding, electrostatic interactions, and target accessibility, which are critical considerations for the design and interpretation of aptamer-based detection platforms. Moreover, pH-dependent alterations in the charge density of the aptamer’s phosphate backbone can influence its folding and electrostatic interactions with the target protein, potentially affecting binding affinity and aptasensor response.

Our previous study^6^ confirmed that recombinant HSA remains stable and retains aptamer-binding ability across a pH range of 4.0–10.0. While the F8 fragment has not yet been evaluated across such conditions, its effective aptamer recognition in PBS at pH 7.4 suggests sufficient stability under physiological pH. Future work will investigate the performance of F8 under broader urinary pH conditions. While our LC–MS/MS analysis confirmed the identity of the F8 fragment with 96% sequence coverage, further validation using clinical urine samples and cross-reactivity tests with abundant urinary proteins such as immunoglobulin light chains and hemoglobin will be pursued to confirm aptamer specificity under complex conditions.

Although this study focused on aptamer binding and structural stability, we acknowledge that future work should include comparative binding assays with known HSA ligands such as warfarin, ibuprofen, and the neonatal Fc receptor (FcRn) to fully validate the functional integrity of recombinant HSA and its fragments. In addition to experimental validation, our previous molecular dynamics studies have revealed that the F8 fragment exhibits the highest conformational stability among HSA-derived peptides and forms more stable interactions with graphene quantum dot–aptamer (GQDA) complexes compared to intact HSA or other fragments.^25,26^ This enhanced structural integrity and binding preference suggest that F8 may retain aptamer affinity even in the presence of diverse interfering substances in complex urine matrices, supporting its potential diagnostic robustness in real-world applications.

## Conclusion

This study demonstrates the enzymatic fragmentation of human serum albumin (HSA) and the aptamer-based detection of resulting fragments using a fluorescence-labeled biosensor. A consistent ∼10 kDa fragment was detected across protease conditions and identified as 96% homologous to the F8 fragment previously reported in patient urine. Considering the presence of both glycosylated and nonglycosylated HSA in clinical samples, we evaluated aptamer binding to recombinant HSA expressed in *E. coli* and rice, as well as recombinant F8.

Despite some aggregation in *E. coli*—expressed proteins, rF8 and rHSA showed higher aptamer signal intensities compared to rice-derived rHSA, suggesting expression system—dependent differences in folding and epitope exposure. These results warrant further investigation using high-resolution tools such as circular dichroism (CD) spectroscopy, NMR, and glycoproteomics to probe structural and post-translational differences.

The aptasensor platform expands on a previously validated system for albumin and glycated albumin detection, which holds ISO 13485 certification and is currently under regulatory review by the Thai FDA. These findings support its potential for clinical translation, particularly in scenarios involving nonfresh urine, where albumin degradation is common. Nonetheless, biological variability, including urine composition, protease activity, and disease heterogeneity, may affect diagnostic performance. In the event of reduced aptamer binding under extreme conditions, future development may involve chemically stabilized aptamers or alternative scaffolds (e.g., LNA-aptamers) to preserve functionality, especially in complex or variable sample environments. Despite these promising results, additional high-resolution structural analyses, such as CD spectroscopy, thermal shift assays, or glycoproteomic profiling, are warranted to further confirm protein integrity and folding, particularly for translational applications. Future multicenter clinical validation using diverse patient-derived samples will be critical to establish robustness and real-world applicability.

## Supplementary Material



## Notes

Figure S1, showing the digestion of the pET17b-HSA plasmid using NdeI and *Eco*RI and the PCR amplification of the F8 gene (291 bp); Figure S2, presenting densitometric analysis of SDS–PAGE band intensities for recombinant HSA (67.5 kDa) and F8 (11.0 kDa); Figure S3, illustrating the estimated diameters of HSA and F8 structures (70–80 Å and 30–40 Å, respectively) using Visual Molecular Dynamics (VMD); Figure S4, predicted disulfide bond topology of the F8 fragment, represented by VMD. Table S1, detailing peptide matches to the F8 sequence from in-gel digestion and LC–MS/MS analysis, with 96% sequence coverage; and Table S2, summarizing the calculated purities of rHSA and rF8 based on band intensities from SDS-PAGE. Table S3, raw fluorescence data (RFU) and standard deviations for aptasensor binding to rHSA (expressed in *E. coli* and rice) and rF8, measured in triplicate (PDF)

## Abbreviations

HSA: human serum albumin
rHSA: recombinant human serum albumin
F8: fragment 8 of HSA (residues 496–585)
CKD: chronic kidney disease
GO: graphene oxide
DLS: dynamic light scattering
PBS: phosphate-buffered saline
IPTG: isopropyl β-d-1-thiogalactopyranoside
SDS-PAGE: sodium dodecyl sulfate-polyacrylamide gel electrophoresis
LC–MS/MS: liquid chromatography–tandem mass spectrometry
PdI: polydispersity index
LOD: limit of detection
FA: formic acid
ACN: acetonitrile
TFA: trifluoroacetic acid
TCEP: tris­(2-carboxyethyl)­phosphine.
