# Supplementary material for: Analytical and Structural Evaluation of Recombinant Human Serum Albumin and Fragment F8 for Aptamer-Based Urinary Biomarker Detection
Source: ACS Omega. 2025 Jul 14;10(28):30935–43. doi: 10.1021/acsomega.5c03518 (PMC12290929; doi:10.1021/acsomega.5c03518)
Supplement: Supplementary file 1 [file ao5c03518_si_001.pdf]

Supplementary Information

Analytical and Structural Evaluation of Recombinant Human Serum Albumin and Fragment F8 for Aptamer-Based Urinary Biomarker Detection

Chanya Archapraditkul[1], Jarunee Vanichtanankul[2], Thanaya Saeyang[2], Wireeya Chawjiraphan[3], Poramin Boonbanjong[4], Prapasiri Pongprayoon[1,5]*, Deanpen Japrung[3]*

[1]Faculty of Science, Department of Chemistry, Kasetsart University, Chatuchak, Bangkok 10900, Thailand

[2]National Center for Genetic Engineering and Biotechnology (BIOTEC), National Science and Technology Development Agency (NSTDA), Thailand Science Park, Pathumthani 12120, Thailand

[3]National Nanotechnology Center (NANOTEC), National Science and Technology Development Agency (NSTDA), Thailand Science Park, Pathumthani 12120, Thailand

[4]Program in Translational Medicine, Chakri Naruebodindra Medical Institute, Faculty of Medicine Ramathibodi Hospital, Mahidol University, Samut Prakan, 10540, Thailand.

[5]Center for Advanced Studies in Nanotechnology for Chemical, Food and Agricultural Industries, KU Institute for Advanced Studies, Kasetsart University, Bangkok 10900, Thailand

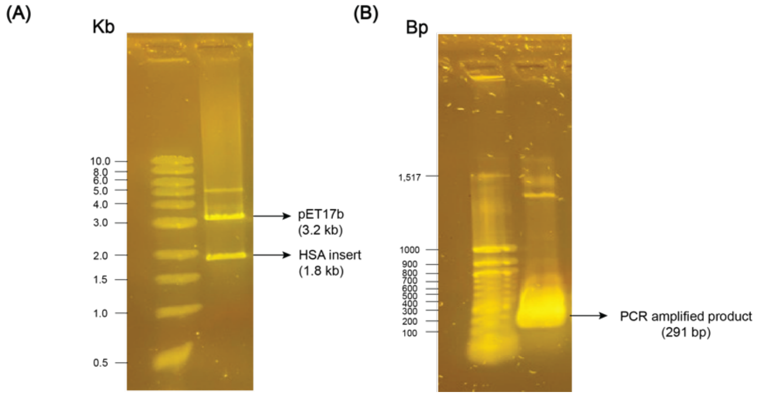

Figure S1. (A) Digestion of the pET17b-HSA plasmid with NdeI and EcoRI restriction enzymes, confirming the presence of expected vector fragments. (B) Agarose gel electrophoresis showing amplification of the F8 gene by PCR, with the product corresponding to the expected size of 291 bp.

Table S1. Peptide matches to the F8 sequence (residues 496–585 of HSA) identified from in-gel tryptic digestion and LC–MS/MS analysis. Bolded peptide entries indicate an expectation score (Expect) < 0.05, suggesting statistically significant matches. The analysis revealed high-confidence peptide coverage across the F8 sequence, resulting in an overall sequence coverage of 96%.

Query	Dupes	Observed	Mr(expt)	Mr(calc)	Delta	M	Score	Expect	Rank	U	Peptide
**1443**	**1**	**659.4389**	**658.4316**	**658.365**	**0.0666**	**0**	**31**	**0.00084**	**1**	**U**	**A.SQAALGL.-**
**1445**	**1**	**659.4447**	**658.4375**	**658.365**	**0.0725**	**0**	**33**	**0.00051**	**1**	**U**	**K.LVAASQA.A**
**1677**	**10**	**386.2969**	**770.5792**	**770.4902**	**0.089**	**0**	**22**	**0.0068**	**1**	**U**	**T.ALVELVK.H**
1715		394.2843	786.5541	787.444	-0.8898	0	1	0.72	1	U	K.KQTALVE.L
1719		394.2852	786.5558	786.4963	0.0595	0	4	0.42	1	U	I.KKQTALV.E
1726	1	789.5524	788.5452	787.444	1.1012	0	13	0.054	1	U	K.KQTALVE.L
1742		399.2095	796.4044	797.3596	-0.9552	0	4	0.37	1	U	D.DFAAFVE.K
1805		414.1872	1652.7195	1652.7471	-0.0276	0	4	0.4	1	U	V.PKEFNAETFTFHAD.I
1808		414.1875	1652.7209	1653.0301	-0.3092	0	0	1	1	U	E.RQIKKQTALVELVK.H
1910	1	421.8075	841.6005	842.4861	-0.8856	0	4	0.41	1	U	K.LVAASQAAL.G
**1926**	**3**	**843.5788**	**842.5715**	**842.4861**	**0.0854**	**0**	**64**	**3.60E-07**	**1**	**U**	**K.LVAASQAAL.G**
1943		425.7614	849.5082	849.4021	0.1061	0	1	0.85	1	U	T.FTFHADI.C
**2018**	**1**	**900.6165**	**899.6093**	**899.5076**	**0.1017**	**0**	**40**	**9.70E-05**	**1**	**U**	**K.LVAASQAALG.L**
**2020**	**1**	**450.8126**	**899.6107**	**899.5076**	**0.1031**	**0**	**14**	**0.04**	**1**	**U**	**K.LVAASQAALG.L**
2056		303.2026	906.5861	907.5491	-0.963	0	6	0.23	1	U	T.ALVELVKH.K
2206		483.7746	965.5347	965.4243	0.1104	0	1	0.76	1	U	F.NAETFTFH.A
2208		484.7808	967.547	967.4433	0.1038	0	3	0.53	1	U	T.CFAEEGKK.L
2218		491.8545	981.6945	982.443	-0.7485	0	1	0.81	1	U	L.KAVMDDFAA.F + Oxidation (M)
2277	2	493.3207	984.6268	984.3859	0.241	0	5	0.34	1	U	K.ADDKETCF.A
2320		500.8001	999.5855	998.4015	1.184	0	1	0.86	1	U	D.DKETCFAE.E
**2330**	**63**	**500.8571**	**999.6997**	**999.5964**	**0.1033**	**0**	**41**	**8.10E-05**	**1**	**U**	**K.QTALVELVK.H**
**2341**	**2**	**1000.7074**	**999.7001**	**999.5964**	**0.1037**	**0**	**16**	**0.023**	**1**	**U**	**K.QTALVELVK.H**
2421		335.2253	1002.654	1001.5505	1.1034	0	9	0.14	1	U	T.LSEKERQI.K
2442		509.3268	1016.639	1016.5614	0.0776	0	7	0.21	1	U	L.SEKERQIK.K
**2476**		**518.8095**	**1035.6045**	**1035.4906**	**0.1139**	**0**	**15**	**0.033**	**1**	**U**	**H.ADICTLSEK.E**
**2545**	**4**	**535.7858**	**1069.5571**	**1069.4386**	**0.1185**	**0**	**27**	**0.0018**	**1**	**U**	**K.ETCFAEEGK.K**

2610		362.5689	1084.6848	1085.4852	-0.8004	0	1	0.84	1	U	D.DFAAFVEKC.C
2663	11	564.9108	1127.8071	1127.6914	0.1157	0	70	9.50E-08	1	U	K.KQTALVELVK.H
2669	4	376.9434	1127.8082	1127.6914	0.1168	0	25	0.0029	1	U	K.KQTALVELVK.H
2711	1	575.3707	1148.7268	1148.7393	-0.0125	0	4	0.41	1	U	E.LVKHKPKATK.E
2766	2	595.8907	1189.7669	1189.7547	0.0122	0	2	0.6	1	U	A.LVELVKHKPK.A
2771		398.9208	1193.7406	1193.688	0.0526	0	8	0.15	1	U	V.KHKPKATKEQ.L
2774	1	400.2272	1197.6598	1197.5336	0.1262	0	10	0.11	1	U	K.ETCFAEEGKK.L
2777	2	599.8388	1197.6631	1197.5336	0.1296	0	50	1.00E-05	1	U	K.ETCFAEEGKK.L
2823		616.8711	1231.7277	1230.6932	1.0345	0	2	0.65	1	U	C.TLSEKERQIK.K
2839		631.8722	1261.7298	1261.7064	0.0235	0	1	0.72	1	U	P.KATKEQLKAVM.D + Oxidation (M)
2871		446.6326	1336.8759	1336.6333	0.2426	0	2	0.57	1	U	K.EQLKAVMDDFAA.F
2884	11	679.895	1357.7755	1357.6224	0.1531	0	44	4.50E-05	1	U	K.AVMDDFAAFVEK.C + Oxidation (M)
2906		465.2979	1392.8718	1392.8453	0.0265	0	0	0.92	1	U	I.KKQTALVELVKH.K
2910	3	474.6113	1420.812	1420.6657	0.1463	0	16	0.028	1	U	F.TFHADICTLSEK.E
2917	3	711.4156	1420.8167	1420.6657	0.151	0	41	8.80E-05	1	U	F.TFHADICTLSEK.E
2948		362.4797	1445.8897	1444.6479	1.2418	0	1	0.84	1	U	D.DFAAFVEKCCKA.D
2961		498.6383	1492.8931	1491.7603	1.1328	0	1	0.79	1	U	P.KATKEQLKAVMDD.F + Oxidation (M)
2970	3	500.5999	1498.778	1498.6246	0.1534	0	35	0.00028	1	U	K.ADDKETCFAEEGK.K
2980	5	750.3989	1498.7832	1498.6246	0.1587	0	50	9.90E-06	1	U	K.ADDKETCFAEEGK.K
3004		513.3135	1536.9186	1537.7201	-0.8015	0	0	0.99	1	U	V.PKEFNAETFTFHA.D
3040		528.2645	1581.7717	1582.8065	-1.0348	0	1	0.87	1	U	Q.LKAVMDDFAAFVEK.C
3071	15	543.3024	1626.8854	1626.7195	0.1659	0	50	1.00E-05	1	U	K.ADDKETCFAEEGKK.L
3076	6	814.4504	1626.8863	1626.7195	0.1668	0	63	5.40E-07	1	U	K.ADDKETCFAEEGKK.L
3080	4	407.7289	1626.8867	1626.7195	0.1671	0	4	0.38	1	U	K.ADDKETCFAEEGKK.L
3085		407.7294	1626.8883	1627.7916	-0.9033	0	1	0.8	1	U	T.KEQLKAVMDDFAAF.V + Oxidation (M)
3110		411.7295	1642.8888	1641.6797	1.2091	0	1	0.79	1	U	F.VEKCCKADDKETC.F
3127		423.7131	1690.8232	1689.8719	0.9513	0	2	0.67	1	U	H.ADICTLSEKERQIK.K
3132		439.257	1752.9987	1752.008	0.9908	0	1	0.81	1	U	V.KHKPKATKEQLKAVM.D + Oxidation (M)
3150		396.4128	1977.0277	1975.8478	1.1799	0	2	0.57	1	U	K.AVMDDFAAFVEKCCKAD.D
3190		399.2808	2389.6413	2389.1116	0.5297	0	6	0.27	1	U	A.TKEQLKAVMDDFAAFVEKCC.K
3193		335.0428	2672.2842	2673.0856	-0.8014	0	1	0.89	1	U	V.MDDFAAFVEKCCKADDKETCFA.E + Oxidation (M)
3196		335.0446	2672.2988	2671.4578	0.8409	0	1	0.71	1	U	H.ADICTLSEKERQIKKQTALVELV.K
3199		448.2896	2683.6941	2685.1219	-1.4279	0	2	0.57	1	U	A.VMDDFAAFVEKCCKADDKETCF.A
3209		440.7711	3078.3467	3078.5453	-0.1986	0	1	0.79	1	U	V.KHKPKATKEQLKAVMDDFAAFVEKCC.K
3213		659.4448	3292.1876	3292.6761	-0.4885	0	2	0.64	1	U	N.AETFTFHADICTLSEKERQIKKQTALVE.L

**3214**		335.046	3340.3875	3341.4713	-1.0837	0	2	0.64	**1**	U	E.QLKAVMDDFAAFVEKCCKADDKETCFAE.E + Oxidation (M)

Bold letters refer to the expect score lower than 0.05

Protein sequence coverage: 96%

TYV**PKEFNAETFTFHADICTLSEKERQIKKQTALVELVKHKPKATKEQLKAVMDDF AAFVEKCCKADDKETCFAEEGKKLVAASQAALGL**

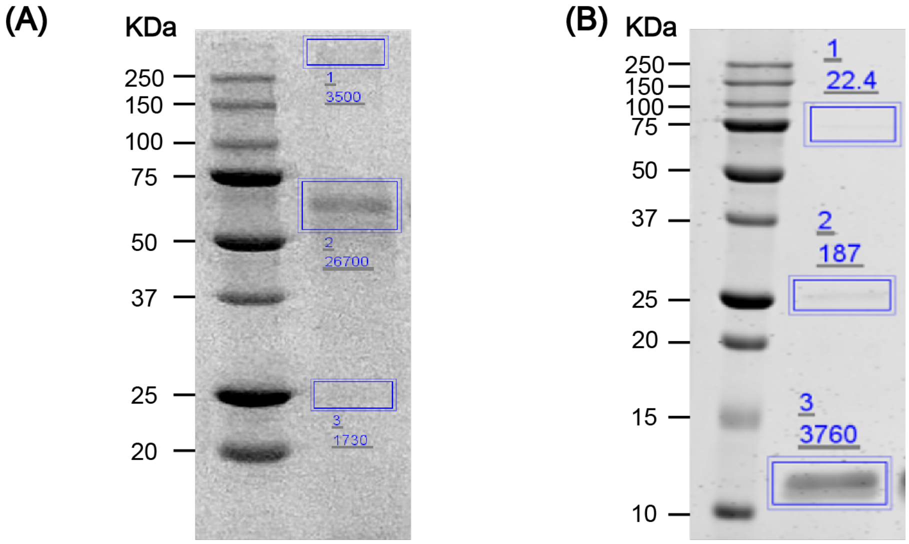

Figure S2. Densitometric analysis of 12% SDS–PAGE band intensities for recombinant HSA (A) and F8 (B). The prominent band corresponding to HSA (67.46 kDa) is marked as band no. 2 in panel A, while the F8 fragment (11.04 kDa) appears as band no. 3 in panel B.

Table S2. Quantitative analysis of recombinant protein purities based on 12% SDS–PAGE band intensity measurements. The signal intensities of individual bands were used to calculate the relative purity percentages of recombinant HSA and F8 proteins.

**Band**	**HSA**		**F8**	
	**Signal**	**% purity**	**Signal**	**% purity**
1	3500		22.4	
2	26700	**83.62042**	187	
3	1730		3760	**94.72464**
Total	31930		3969.4	

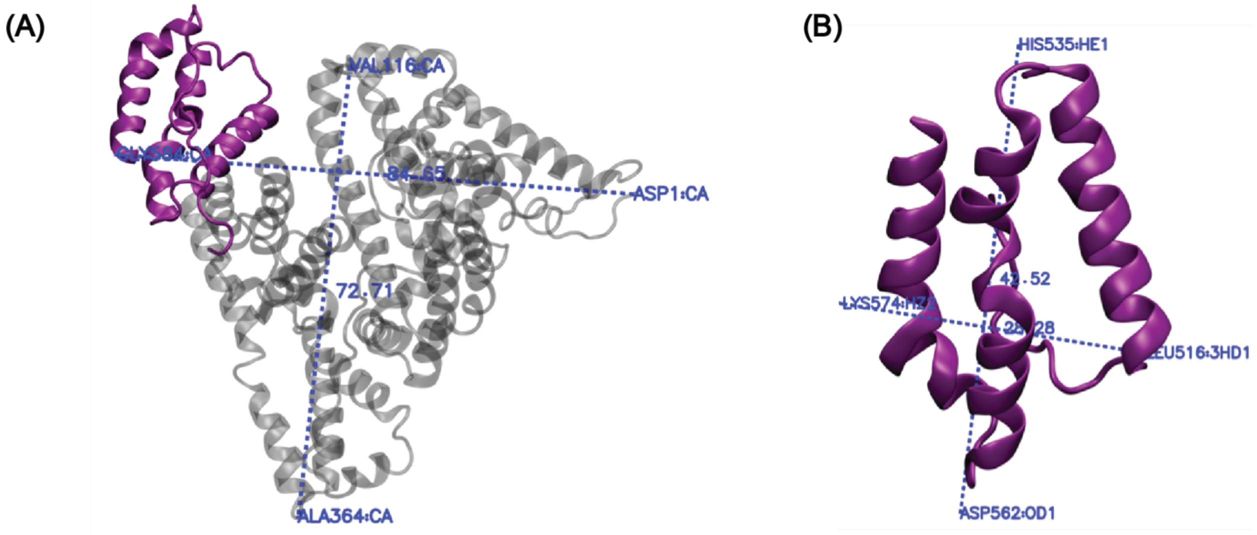

Figure S3. Structural estimation of protein diameters using Visual Molecular Dynamics (VMD). (A) Full-length HSA exhibits an approximate diameter of 70–80 Å (7–8 nm). (B) The F8 fragment shows a reduced diameter of approximately 30–40 Å (3–4 nm).

Table S3. The relative fluorescence unit (RFU) of aptasensor binding rHSA expressed from rice and *E.coli* and rF8 from *E.coli.*

Conc (mg/mL)	**HSA (rice)**		**HSA (*E.coli)***		**F8**	
	RFU	SD	RFU	SD	RFU	SD
1.5	5720.67	54.72	11086.00	19.31	10376.00	30.61
0.75	4156.00	2.00	5400.33	13.50	9223.00	3.61
0.375	1578.00	0.00	856.00	3.00	3887.00	2.08
0.1875	501.33	0.58	218.33	0.58	1149.67	4.04
0.09375	174.67	1.53	157.00	1.00	316.67	1.15
0.046875	152.67	5.51	145.67	1.53	198.33	1.53

0.0234375	127.67	1.15	133.33	2.52	173.00	3.00
0.01171875	132.33	7.02	133.00	1.00	151.33	2.52
0.005859375	122.67	1.15	134.33	3.21	152.33	3.51
0.002929688	129.33	1.53	140.00	1.73	137.33	1.53
0.001464844	117.67	2.08	126.00	2.65	132.00	4.58
0.000732422	122.67	5.69	118.67	3.06	160.00	6.56
0.000366211	121.67	2.08	114.33	2.52	137.00	2.65

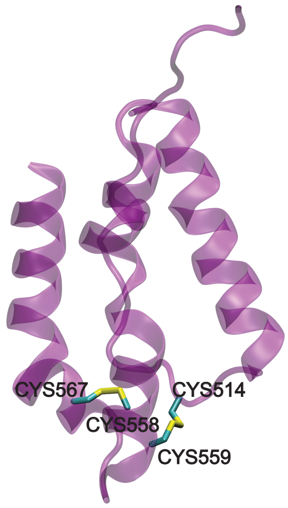

Figure 4. The schematic view of F8 represented by VMD software.
